# Tumor Angiogenesis: Insights and Innovations

**DOI:** 10.1155/2010/132641

**Published:** 2010-04-26

**Authors:** Fernando Nussenbaum, Ira M. Herman

**Affiliations:** ^1^Tulane University School of Medicine, 257 Cherokee st., Unit 2, New Orleans, LA 70118, USA; ^2^Center for Innovations in Wound Healing Research, Tufts University School of Medicine, 150 Harrison Avenue, Boston, MA 02111, USA

## Abstract

Angiogenesis is a vital process resulting in the formation of new blood vessels. It is normally a highly regulated process that occurs during human development, reproduction, and wound repair. However, angiogenesis can also become a fundamental pathogenic process found in cancer and several other diseases. To date, the inhibition of angiogenesis has been researched at both the bench and the bedside. While several studies have found moderate improvements when treating with angiogenesis inhibitors, greater success is being seen when the inhibition of angiogenesis is combined with other traditional forms of available therapy. This review summarizes several important angiogenic factors, examines new research and ongoing clinical trials for such factors, and attempts to explain how this new knowledge may be applied in the fight against cancer and other angiogenic-related diseases.

## 1. Introduction

For over 35 years, scientists have been trying to fully understand the process of both normal and pathogenic angiogeneses, hoping to apply their findings to the world of clinical medicine and therapeutics. Angiogeneses is a critical process involving the formation of new blood vessels from preexisting vessels [1]. Normal angiogeneses is an essential process the body employs during fetal development, wound healing, ovulation, as well as growth and development [2]. Angiogeneses provides developing and healing tissues with vital nutrients and oxygen [3]. When angiogeneses goes awry, pathological problems often ensue. The understanding of normal and pathogenic angiogeneses has been a major focus of both cancer biology and clinical medicine for the past few decades. 

In the past, research in angiogeneses was closely intertwined with cancer biology. The importance of angiogeneses in tumor growth was initially hypothesized in 1971, when Judah Folkman theorized that solid tumors possess limited resources that the many actively proliferating cancer cells fight for. Increased interstitial pressure within the tumor also inhibits the diffusion of metabolites and nutrients essential to the growth and survival of tumor cells [4]. This environment causes tumor cells to induce the sprouting of new blood vessels from the established vasculature, creating a vascular system within the tumor, thus enabling tumor cells to obtain the oxygen and nutrients they need to survive and multiply. Understanding these principles led to the idea that the inhibition of tumor angiogeneses could be a valuable therapy against cancer [1]. This sparked research into the proteins that regulate this process, both angiogeneses inhibitors and promoters. Since that time, many proteins and regulators of angiogeneses have been discovered and their role in the process defined.

Although cancer has traditionally been the most extensively studied angiogenic-dependent disease, several other conditions have also shown a reliance on angiogeneses. Some of these include psoriasis, endometriosis, arthritis, macular degeneration, regional ileitis, and atherosclerosis [5]. The emergence of other diseases connected to angiogeneses has led to increased research on angiogeneses as a whole. Recently, new drugs have been developed that are capable of targeting many of the regulators of angiogeneses [6]. Currently, several drugs have been approved by the FDA for the treatment of angiogeneses-dependent diseases including Avastin for colorectal cancer, Tarceva for lung cancer, and Lucentis for macular degeneration [5, 7]. Many other drugs are in late-stage clinical testing. This review will focus on the current knowledge of angiogeneses in health and disease, some important angiogenic promoters and inhibitors, and ongoing research and developments as they relate to oncology. Increasing the mechanistic understanding of these processes will improve the development of more efficient angiostatic treatments in cancers.

## 2. Normal Blood Vessel Formation

**Figure 1:**
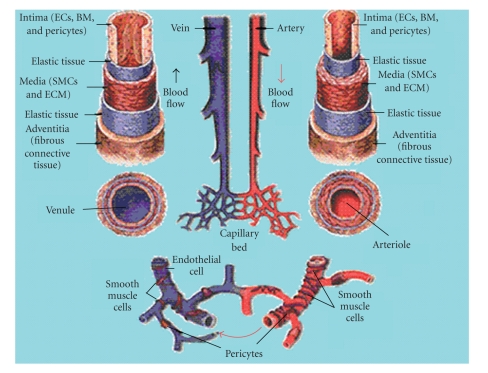
*Blood Vessels*. The cardiovascular system main components include arteries, arterioles, capillaries, venules, and veins. Each vessel has cellular differences from the other types of vessels and this is highlighted above.

The cardiovascular system distributes blood, and thus oxygen and nutrients, throughout the body. The system consists of arteries, arterioles, capillaries, venules, and veins. The microvasculature is considered the portion of the circulatory system composed of the smallest vessels, such as the capillaries, arterioles, and venules. The microvasculature is a very dynamic and complex system, capable of constant change, while the larger blood vessels are more permanent structures with very little plasticity. As illustrated in Figure 1, capillaries are hollow tubes composed of endothelial cells (ECs) which are supported by pericytes. Unlike capillaries, arteries and veins have several distinct layers including the tunica intima, the tunica media, and tunica adventitia in the largest vessels (composition of each detailed in Figure 1). Due to the thickness of these structures, arteries, arterioles, venules, and veins are all considered conduit vessels. Capillaries are the most important vessels in cardiovascular system. The thin walls of these microscopic vessels allow for the exchange of oxygen and nutrients between the blood and tissues [7]. The formation of the initial vascular plexus within each tissue and the formation of the major blood vessels conducting blood to and from the heart are hard wired into the developmental system [8]; these networks are formed independent of oxygen concentration. In contrast, the pattern of capillary (microvasculature) development within each tissue is driven by local oxygen demand, and is therefore unique to each tissue [8]. 

Blood vessels comprising the microvasculature are formed in adults via two different mechanisms: vasculogenesis and angiogeneses. Both processes normally occur during embryonic development; however, special circumstances allow these processes to be initiated during adult life. Vasculogenesis is the de novo formation of ECs from angioblasts. This process helps form a primitive vascular labyrinth of small capillaries [9]. Angiogeneses is the process in which ECs sprout from preexisting blood vessels. The ECs then migrate and proliferate to form a cord-like structure.

### 2.1. Vasculogenesis: Current Concepts and Challenges

The establishment of fetal vasculature begins with hemangioblasts, primitive cells of mesodermal origin [10]. Hemangioblasts help form “blood islands”, clusters of cells that have a designated spatial arrangement that facilitates their function. Hematopoietic stem cells (HSCs), which later become hematopoietic cells, are found at the center of these islands. Angioblasts, cells that differentiate into ECs, are found at the periphery of the blood islands [11]. The adult stem cells found within bone marrow (instead of blood islands) were discovered to contain much greater plasticity than originally thought, and are now considered multipotent adult progenitor cells (MAPCs) [12]. MAPCs are capable of differentiating into ECs when removed from bone marrow and cultured on fibronectin with vascular endothelial growth factor-A (VEGF-A) [13]. In addition, MAPCs are capable of differentiating into skeletal muscle, cardiac muscle, and vascular endothelium after bone marrow transplantation [12].

**Figure 2:**
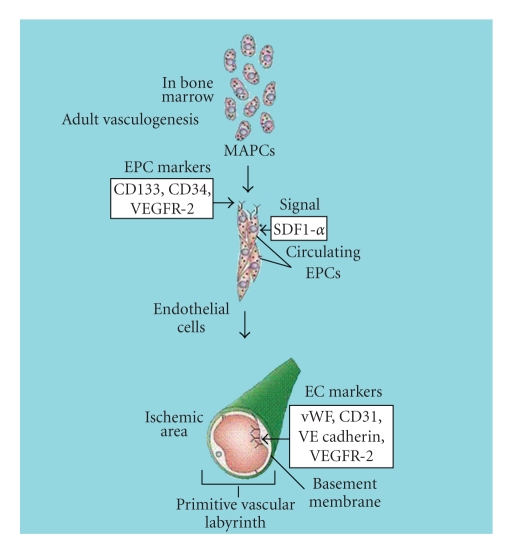
*Adult Vasculogenesis.* The figure illustrates the process where MAPCs become angioblasts, then circulating EPCs and ECs as part of the primitive vascular labyrinth.

As demonstrated in Figure 2, it is currently believed that vasculogenesis originates when MAPCs in bone marrow differentiate into early endothelial progenitor cells (EPCs) [13, 14]. As MAPCs evolve into EPCs, they gain hematopoietic and endothelial lineage-specific markers such as VEGF receptor-2 (VEGFR-2) and CD34 [15, 16]. EPCs in the bone marrow remain undifferentiated in one of two zones. The first zone is known as the vascular zone, and it consists of EPCs in either the S phase or G2M phase of the cell cycle. These cells are capable of differentiating and entering peripheral circulation upon receiving the correct signals [14, 17]. The second zone is known as the osteoblastic zone, where EPCs are maintained in the G0 phase of the cell cycle. These cells are not actively dividing, and therefore not readily available for release into circulation [14, 18]. The balance between these two functional compartments is maintained by cytokines present in the bone marrow's extracellular matrix (ECM) and on bone marrow stromal cells [19]. The bone marrow stromal cells and ECM preserve levels of cytokines bound to either ECM proteins or cell membranes. The cytokines can be cleaved by proteinases and activated. To illustrate, matrix metalloproteinases (MMPs) help mediate the digestion of ECM which leads to the release of membrane-bound cytokines. This allows the release of VEGF-A, an important regulator of both angiogeneses and vasculogenesis [20]. Evidence has suggested that MMP-9 is capable of mobilizing EPCs to enter the vascular zone and eventually be released into the peripheral circulation [14, 17]. Malignant transformation, tissue injury, or ischemia can induce systemic release of VEGF-A, activating bone marrow progenitor stem cells [15]. 

A recent study verified altered levels of MMP-9 and VEGF-A in patients with early-stage breast and colorectal cancer when compared to normal patients [57]. Healthy volunteers showed VEGF-A plasma levels averaging 37.6 *μ*g/mL, while the breast cancer patients had average plasma concentrations of 52.9 *μ*g/mL and colorectal cancer patients plasma concentrations averaged 109.6 *μ*g/mL. MMP-9 plasma concentrations for healthy volunteers averaged 169 ng/mL, while breast cancer patients had plasma concentrations averaging 237.8 ng/mL and colorectal cancer patients averaged 370.1 ng/mL. The patients underwent surgical resection of their primary tumors and the levels of MMP-9 and VEGF-A were measured between 7 and 8 weeks postsurgery. These same patients saw a decrease in their MMP-9 and VEGF-A plasma levels [57]. The breast cancer patients saw a decrease in the average VEGF-A plasma concentration from 52.9 *μ*g/mL to 43.8 *μ*g/mL and their MMP-9 levels dropped from an average of 237.8 ng/mL to 109.6 ng/mL. The colorectal cancer patients saw similar changes after their tumors were removed with their average VEGF-A plasma levels dropping from 109.6 *μ*g/mL to 57.6 *μ*g/mL and their MMP-9 plasma levels going from 370.1 ng/mL to 190.3 ng/mL [57]. This evidence shows the direct effect that malignancy can have on factors important to both vasculogenesis and angiogeneses. 

During the process of vasculogenesis, EPCs that enter the peripheral circulation migrate to the areas where the vasculature will be established. The chemokine stromal-cell-derived factor-1*α* (SDF-1*α*) helps mediate the migration of many stem cells, including EPCs. SDF-1*α* is upregulated during hypoxic conditions due to increased levels of VEGF-1 [56]. Once released, SDF-1*α* acts as a key homing signal, helping to guide EPCs to areas of ischemia [58, 59]. The guided cells are still considered early EPCs because they are positive for CD133, CD34, and VEGFR-2 (as demonstrated in Figure 2) [19]. While in circulation, the EPCs continue to differentiate. They begin this process by losing the CD133 marker, and gaining EC-specific markers such as von Willebrand Factor (vWF), CD31, and VE cadherin [60]. EPCs normally compose approximately 0.002% of the mononuclear cell fraction of blood [61]. However, if neovascularization is required, vasculogenic stimuli are released, increasing the circulating concentration of EPCs [62–64]. Clinical trials have demonstrated this phenomenon using patients who either suffered burns or underwent coronary artery bypass graft surgery [65]. Both patient groups saw a 50-fold increase in EPC levels within the first 6 hours after the initiating event, with a return to basal levels within 72 hours. It is believed that this transient increase in EPC levels caused by the vascular and tissue trauma induces the release of several cytokines, including VEGF, promoting EPC mobilization and the initiation of vasculogenesis [65]. 

After EPCs arrive and enter the target tissue, some continue their differentiation into mature ECs [19]. It is thought that the cells that do not differentiate into mature ECs act as a source of proangiogenic cytokines [19]. The maturation into ECs is marked by the loss of the CD34 marker on the cell surface.

Vasculogenesis that occurs during postfetal life in response to angiogenic cytokines has a few key differences from the vasculogenesis that occurs during embryonic life. One major difference is that the formation of the initial vascular plexus in embryonic life is not driven by insufficient oxygen like the vasculogenesis that occurs later in life [8]. An imbalance in oxygen supply and demand can cause hypoxia resulting in an induction of cytokine production or release from cells throughout the body. One such cytokine released during hypoxic conditions is VEGF. After VEGF is released, it binds to VEGF receptors on ECs. This leads to the activation of signal transduction pathways capable of stimulating both angiogeneses and vasculogenesis. The hypoxic conditions seen in tumor cells have been studied and factors associated with vasculogenesis and angiogeneses have been monitored to detail their relationships. Many of these factors have been listed in Table 1. 

As discussed above, Zaman's work on colorectal and breast cancer patients specifically showed increased concentrations in VEGF-A and MMP-9 plasma levels due to malignancy. These levels are reduced after the tumor is removed, and thus levels of angiogeneses and vasculogenesis are decreased. Another difference in the two types of vasculogenesis is that most of the cells recruited to sites of vasculogenesis during postembryonic life are inflammatory cells and are not incorporated into the new capillaries or remodeling arteries. The postembryonic form of vasculogenesis is much more similar to angiogeneses then the embryonic form of vasculogenesis and this is illustrated by the similar factors that are vital to postembryonic vasculogenesis and angiogeneses.

### 2.2. Angiogeneses: Current Concepts, Known Factors, and Challenges

**Figure 3:**
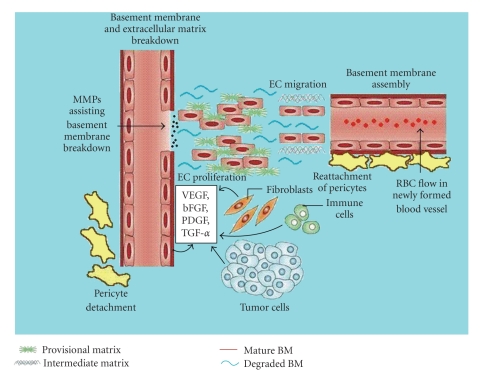
*Tumor Influenced Angiogeneses*. The stepwise process of angiogeneses begins with ECM and BM breakdown, followed by EC proliferation, EC migration and finally re-formation of stable blood vessel. Tumor cells will secrete a variety of factors to ensure that the new blood vessels formed are fed directly to the tumor tissue.

In contrast to vasculogenesis, angiogeneses is the expansion of preexisting vasculature, such as a vascular labyrinth of capillaries, by means of budding and branching into a functional capillary bed which is illustrated in Figure 3. This normally occurs in very organized manner forming what is known as primary vascular trees [66]. Like vasculogenesis, angiogeneses occurs most often during embryonic development; however, it can also occur in adult life in response to specific stimulations. Nonpathogenic angiogeneses can be seen in adults during the ovarian cycle, in skeletal and cardiac muscle during times of exercise and training, as well as during the process of wound healing [3].

The process of angiogeneses is very closely regulated. Stimulation of angiogeneses occurs by growth factors such as VEGF and FGF (see Figure 3, Table 1). New blood vessel formation actually begins with the removal of mural cells (pericytes) from preexisting blood vessels. The absence of these pericytes initiates the degradation of the EC basement membrane and extracellular matrix, a process which is aided by MMPs [67]. 

As the basement membrane and extracellular matrix are being degraded, ECs begin proliferating and migrating with the help of soluble growth factors. The ECs will continue to grow until they form an unstable microvessel. Following the formation of this small blood vessel, mesenchymal cells are recruited to the vessel, where they are subsequently differentiated into pericytes. After differentiation, cell-cell contact between pericytes and ECs occurs. Stable blood vessels are then formed and blood flow can be established. This process of angiogeneses is visualized in Figure 3. Vessels made from ECs not covered with pericytes are unstable, and undergo regression [3, 21]. There are many known factors that help regulate angiogeneses. Some of the known factors are touched upon in Table 1 and discussed in further detail below. 

### 2.3. Angiogenic Promoters: Current Research and Clinical Implications

#### 2.3.1. Vascular Endothelial Growth Factor

VEGF is an important regulator of both vasculogenesis and angiogeneses [21]. Several cell types including fibroblasts, ECs, and keratinocytes release a small amount of VEGF throughout life. The loss of a VEGF allele always results in embryonic lethality [35]. Increased levels of VEGF are seen when angiogeneses is necessary, such as during active wound healing [68]. 

There are currently six known monomers of VEGF that arise from alternative splicing of a single gene with eight exons. The documented isoforms contain 121, 145, 165, 183, 189 or 206 amino acids [69–72]. Some of these isoforms remain associated with cells or membranes, while others are released extracellularly. Despite these differences, all of them have identical biological activities [73]. 

VEGF interacts with two different receptor tyrosine kinases, VEGFR-1 (Flt-1) and VEGFR-2 (Flk-1), to alter angiogeneses. VEGFR-1 interacts very strongly with VEGF, but this interaction plays a minor role in the events of angiogeneses [74]. The interaction of VEGFR-2 with VEGF is a major contributor to the mitogenic, chemotactic, angiogenic, and increased permeability effects of VEGF. VEGFR-2 expression has been observed on both endothelial and hematopoietic precursors [22]. 

Experiments have shown that VEGF has the ability to stimulate microvascular EC proliferation [22, 23]. VEGF is also capable of enhancing EC migration [24], inhibiting EC apoptosis [26], and inducing the growth of new capillaries from preexisting vasculature [25]. VEGF helps induce EC migration and sprouting by upregulating integrin receptors *α*V*β*3, *α*1*β*1, and *α*1*β*2 (discussed below) [27, 75]. VEGF also helps activate MMPs, an important step in the initial stages of angiogeneses. 

VEGF is upregulated during hypoxic conditions via the following mechanism, which involves hypoxia-inducible factor-1 (HIF-1), a protein released during oxygen stress [76]. Responses to ischemia are mostly regulated by cells within the ischemic area monitoring oxygen concentrations. After sensing reduced concentrations, these cells increase their expression of genes encoding vascular growth factors. At the heart of this pathway is HIF-1, a heterodimeric transcription factor composed of the constitutively expressed HIF-1*β*, and the oxygen-sensitive HIF-1*α* subunits [77, 78]. Although HIF-1*α* is also constitutively expressed, it is broken down within adequately oxygenated and perfused tissue. When tissues are deprived of oxygen, the breakdown of HIF-1*α* is inhibited. HIF-1*α* begins to build up and eventually dimerizes with HIF-1*β*. This complex then binds to DNA, helps recruit coactivators, and activates transcription of its target genes [79]. This system induces expression of several vasculogenic and angiogenic growth factors including VEGF and PLG in response to hypoxia [80]. HIF-1 is also required for expression of VEGFR-1 on EPCs in the bone marrow and the chemotactic migration of EPCs towards a VEGF gradient [79]. 

The use of anti-VEGF drugs has been applied to many different fields of medicine. The American Academy of Ophthalmology recently published a report stating that the use of anti-VEGF pharmacotherapy is a safe and effective treatment for neovascular age-related macular degeneration (AMD) [81]. Anti-VEGF signaling pathway drugs have also been tested in a large number of clinical and laboratory studies aimed at preventing angiogeneses associated with cancer. Some of these drugs target VEGF (Avastin), while others target the VEGFRs (Nexavar and Sutent) [82]. Although these drugs have seen dramatic results in animal models [83–85], the results in many of the clinical trials have been mixed [86]. There have been clinical trials which show as many as 94% of invasive carcinomas and 88% of in situ carcinomas having a complete response [87]. These same patients saw no recurrence during the five-year followup [87]. However, many other angiogeneses inhibitors targeting VEGF signaling pathways have failed to produce the same long-term responses in a majority of their patients [86, 88, 89]. A short-term response of either tumor stasis or increased survival was normally observed in these patients [90]; however after the initial benefit, most patients experienced tumor growth after several months [90]. These contradictory results have changed the philosophy on the resistance of tumors to antiangiogenic treatments, as well as the vascular makeup thought to be associated with the blood vessels that support tumors. These concepts will be further explored in the future directions section. 

Inducing neovascularization in ischemic diseases such as chronic wounds and myocardial infarction is also a very active area of research and is leading to a greater understanding of how VEGF works in the body. Several groups have attempted to induce angiogeneses in ischemic tissue through local delivery of the VEGF gene or protein. In clinical trials, local delivery of pro-VEGF growth factors has induced modest levels of neovascularization in ischemic tissue [91]. However, the amount of angiogeneses was inadequate as a monotherapeutic treatment [91]. This information is also valuable for the field of tumor angiogeneses. It is evident that although VEGF is an important part of angiogeneses, controlling its level alone is not enough to regulate angiogeneses in normal tissues or tumors.

#### 2.3.2. Fibroblast Growth Factor

Another important set of proteins mediating angiogeneses is the FGF family. FGFs are soluble growth factors that come in an acidic (aFGF) and basic (bFGF) variety. Both types consist of widespread polypeptides that are powerful inducers of EC migration, proliferation, and microvessel tube formation (Figure 3) [3, 30]. While VEGF is a specific mitogen for ECs, the same cannot be said for FGF. FGF is pleiotropic; it stimulates proliferation in nearly all cells derived from embryonic mesoderm or neuroectoderm [92]. Recent evidence suggests that FGF does not play a major role in generalized angiogeneses in vivo, as mice deficient in both forms of FGF underwent normal development [31]. Instead, FGF seems to be more important in the remodeling of damaged blood vessels [31] which can occur during both wound healing and tumor angiogeneses. FGF is generally found in the cytoplasm of cells or bound to heparin in the ECM [93, 94]. After tissue damage occurs, it appears that FGF is released from the damaged cell(s). This local release of FGF is thought to help promote angiogeneses at the site of the damaged vessel. 

One group recently studied the role of FGF in vascular integrity and human saphenous vein ECs in vitro by disrupting FGF signaling in bovine aortic endothelial cells [95]. They also disrupted the FGF signaling pathway in adult mouse and rat ECs in vivo using soluble FGF traps or a dominant inhibitor of all FGF receptors [95]. Inhibition of this signaling pathway led to a loss of function in the adherens and tight junctions, which caused the loss of EC's, severe impairment of the endothelial barrier function, and finally, disintegration of the vasculature [95]. This experiment showed another possible mechanism for the inhibition of angiogeneses. A targeted approach could possibly allow the breakdown of specific vasculature. In addition to this study, another possible inhibitor of both FGF and VEGF was examined. A plasma glycoprotein, Beta-2 glycoprotein-1, was found to have inhibitory effects on Human Umbilical Vein Endothelial Cells (HUVEC) proliferation, migration, and tubule formation in a dose-dependent manner [96]. This was accomplished by the downregulation of VEGFR-2, the main mediator of angiogenic signals from VEGF which is also activated by bFGF [96]. When low doses of Beta-2 glycoprotein-1 were applied, HUVEC proliferation was decreased by 11.5% [96]. As this dosage was increased, proliferation continued to decrease until the highest dose, which displayed a 68.9% reduction in proliferation [96].

#### 2.3.3. Tie Receptors

The Tie receptors are a family of tyrosine kinases expressed by ECs that mimic the behavior of VEGF receptors [33, 97]. To date, Tie1 and Tie2 have been identified, and their mechanisms of action studied. Genetically altered mice bred without either receptor underwent normal vasculogenesis, but their ECs lacked normal integrity causing the mice to die from widespread edema and hemorrhage due to a lack of adequate angiogeneses [98]. While both Tie1 and Tie2 receptors are important for vascular integrity [32, 99, 100], only Tie2 appears to be vital to vascular sprouting and branching occurring during angiogeneses [3]. 

Tie Receptor inhibitors have recently been produced in the lab. A research group has developed several Tie inhibitors and tested them in vitro to find one with the best selectivity, potency, and pharmacokinetic parameters [101]. Although preliminary studies for Tie2 inhibitors have begun, further animal and possibly clinical trials still remain.

#### 2.3.4. Angiopoietins

Angiopoietins are protein growth factors that act as ligands for the Tie Receptors on ECs [21]. There are two important angiopoietins that play a role in angiogeneses, Ang-1 and Ang-2. Ang-1 is a well-characterized regulator of angiogeneses. It is an important agonist of and ligand for Tie2 receptors [32]. Experiments have shown that mice that lack Ang-1 or Tie2 receptors will develop normal primary vasculature, but will eventually die because vascular remodeling was never completed [32, 102]. The interaction between Ang-1 and Tie2 is important in angiogeneses as it helps recruit pericytes to newly created blood vessels, increasing the stability of the new vasculature and making it less permeable [32]. Ang-1 also helps induce formation of capillary sprouts and promote survival of ECs [21]. Overexpression of Ang-1 in transgenic mice led to a greater number of blood vessels, with larger diameters, and more vascular branches [34], illustrating the important role Ang-1 plays in vascular sprout formation. 

Preliminary studies have been completed to determine whether Ang-1 inhibitors would be an effective method for reducing levels of angiogeneses. A recombinant human Ang-1 antisense strand was made and used to inhibit Ang-1 expression levels in mice with implanted tumor tissue [103]. Microvascular density in the antisense Ang-1-treated group averaged 6.02 vessels/mm in implanted tumor tissue while the group not receiving the antisense strand averaged 8.44 vessels/mm in the implanted tumor tissue [103]. The group concluded that although Ang-1 inhibitors may help reduce the levels of angiogeneses, it was unlikely that Ang-1 inhibitors alone would be an effective method to inhibit pathogenic angiogeneses. The study suggested that several angiogeneses modulators would need to be inhibited simultaneously to have a noticeable effect on angiogeneses [103]. 

The role of Ang-2 is more complicated than that of Ang-1. It appears that Ang-2 is at least a partial antagonist of Tie2, resulting in pericyte loss [35]. The result of this pericyte loss is destabilization of the blood vessel. Under normal conditions this helps prevent excessive angiogeneses. However, the destabilization also allows newly formed blood vessels to be more plastic. These destabilized vessels show increased endothelial sprouting and tube formation in the presence of VEGF [36]. This fact has made Ang-2 blockers an important area of study for tumor angiogeneses inhibition. A recent study created Ang-2-selective peptide-Fc fusion proteins and antibodies; this enabled a controlled pharmacological inactivation of endogenous Ang-2, allowing the group to study Ang-2 inactivation without using the lethal knockout approach [104]. The use of these inhibitors demonstrated tumor inhibition in mice as well as corneal angiogeneses inhibition in rats [104]. Although the study demonstrated the properties of Ang-2 in vivo and gave therapeutic possibilities to Ang-2 inhibitors, many questions still remain. How Ang-2 inhibition blocks EC proliferation is still not completely understood. In addition, it is unknown if inhibition of both Ang-1 and Ang-2 would further reduce angiogeneses or if blocking one will offset the inhibition of the other. Additionally, these studies have only been completed using animal models, and we can only hope that a similar effect will be seen in humans.

Despite the current problems understanding Ang-2, the selective inhibition of Ang-2 could have a very high clinical value. Studies have demonstrated that Ang-2 upregulation is seen in several diseases that involve pathogenic angiogeneses including cancer, macular degeneration, rheumatoid arthritis, osteoarthritis, and psoriasis [105–107]. One recent study found that mRNA levels of Ang-2 in metastatic liver cells and lymph nodes are between 1.5 and 2 times greater than normal levels [108]. 

#### 2.3.5. Platelet-Derived Growth Factor

PDGF is another important signaling molecule with several different roles in angiogeneses. Although originally purified from platelets, it has also been identified in fibroblasts, astrocytes, ECs, and several other cell types [38]. To date, both hetero and homodimeric versions of PDGF (PDGF-AA, -BB, or -AB) have been studied. Capillary ECs express PDGF-BB receptors, and when the receptors are stimulated, increased DNA synthesis and angiogenic sprouting can be seen in vitro (Figure 3) [38]. Although pericytes are initially recruited to growing microvessels independently of PDGF, pericyte proliferation and migration at a growing blood vessel is enhanced by interaction with PDGF [109]. Mice bred without PDGF-BB or its receptor exhibited a large increase in the permeability of their blood vessels and died prenatally [110]. The interaction of PDGF with its receptor on pericytes increases the expression of Ang-1. This increase in Ang-1 leads to a signaling cascade that helps establish the interaction between pericytes and ECs [97]. This interaction is important for maintaining the stability of newly formed capillary walls [3], a vital part of new blood vessel formation. 

It has been hypothesized that blocking PDGF from interacting with its receptor will reduce the stability of the growing capillaries rendering them incapable of delivering nutrients to the cancer cells [111]. Inhibition of PDGF has been attempted with compounds such as CP-673,451 [111]. Rat glioblastomas treated with CP-673,451 showed a 47% decrease in microvascular density and a 55% decrease in tumor growth. Although a decrease in tumor growth and angiogeneses has occurred through the treatment of cancers with CP-673,451 during in vitro and in vivo animal studies, clinical trials have not been performed to demonstrate its efficacy in humans. Another recent study indicated that lycopene, a carotenoid found in tomatoes, may inhibit PDGF-BB-induced signaling [112] reducing levels of unwanted angiogeneses.

#### 2.3.6. Transforming Growth Factor-Beta

Transforming Growth Factor-Betas (TFG-*β*) are a family of homodimeric cytokines that help control many different processes in the body, including angiogeneses. TGF-*β*'s are normally found in the ECM of many different cells types [113, 114]. Within the microvasculature, both ECs and pericytes produce and display receptors for TGF-*β* [99], illustrating the variety of cells capable TGF-*β* expression. To date, both pro- and antiangiogenic properties have been ascribed to TGF-*β*. At low doses TGF-*β* helps initiate the angiogenic switch by upregulating angiogenic factors and proteinases. However, at high doses TGF-*β* inhibits EC growth, promotes basement membrane reformation, and stimulates SMC's differentiation and recruitment [39]. Genetic studies in mice have shown that the loss of TGF-*β* leads to leaky vessels lacking structural integrity leading to premature death [42]. Stimulation of angiogeneses through TGF-*β* is mostly via indirect mechanisms. TGF-*β* signals inflammatory mediators to the site of angiogeneses, where inflammatory cells release proangiogenic factors such as VEGF, FGF, and PDGF [40, 41].

Recent phase I/II clinical trials attempting to use TGF-*β* inhibitors have been completed. These studies employed a TGF-*β* antisense oligonucleotide, termed AP12009, as a treatment for patients with malignant gliomas [115]. Despite the late stage of the glioma in the patient population, positive efficacy results were observed. Two of the 24 patients saw complete remission of their disease after treatment and remained cancer free 4.5 years after completion of the trial [115]. Seven of the 24 patients also found their disease stabilized after beginning treatment, a larger number compared to controls in other clinical trials [115]. In addition, the median survival time of the AP12009-treated patients was longer than the controls reported by recent literature [115]. After the initial positive results of this trial, a Phase IIB trial was initiated and is still ongoing [115]. These results indicate that the targeted inhibition of TGF-*β* may provide an excellent mechanism to reduce unwanted angiogeneses in a variety of diseases.

#### 2.3.7. Integrins

Integrins are heterodimeric cell surface receptors for ECM proteins that also play a role in cell-cell attachment. They contain various *α*-and *β*-subunits, with over 20 different combinations of subunits known. Integrins are important regulators for many different cell processes including both vasculogenesis and angiogeneses [116]. 

Of the integrins, *α*V*β*3 is one of the most extensively studied, and has an important role in angiogeneses. It binds and activates MMP-2 at the tips of growing blood vessels to help break down the ECM [43]. Integrin *α*V*β*3 shows increased expression in vitro when exposed to VEGF [27] and bFGF [117]. Cell attachment, spreading, and migration are all regulated by integrin *α*V*β*3 in vitro [44]. Angiogenic blood vessels near granulation tissue showed much greater levels of *α*V*β*3 than vessels in uninjured normal skin [45]. During wound repair, *α*V*β*3 is localized to the ECs at the ends of the growing vessels [46]. Anti-*α*V*β*3 monoclonal antibodies reduce bFGF-stimulated angiogeneses, demonstrating an important relationship between the two proteins [47].

Several other integrins have also been implicated in angiogeneses. Inhibition of integrin *α*V*β*5 hindered angiogeneses stimulated by VEGF [47]. Abs to collagen receptor integrins (*α*1*β*1 and *α*2*β*1) also reduced VEGF-mediated angiogeneses [75]. A variety of integrins play important roles in angiogeneses including EC adhesion to ECM, protease localization, and increased EC survival [3]. This diversity suggests a number of different integrins; each plays a distinct role that uniquely contributes to the process of angiogeneses. 

There are currently three classes of integrin inhibitors in preclinical and clinical trials. Some of these include a synthetic peptide Cilengitide (a *α*V*β*3/*α*V*β*5 inhibitor), a monoclonal Ab Abergin (a *α*V*β*3 antagonist), and a peptidomimetic compound S247 (a *α*V*β*3/*α*V*β*5) antagonist [118]. Phase I trials using Vitaxin (similar to Abergin) were unsuccessful in reducing tumor growth [119]. A second generation Phase II trial which altered Vitaxin to give it greater affinity for *α*V*β*3 also failed to reduce tumor growth [120, 121]. Phase I and II trials with Cilengitide have also been completed. Although some antitumor effects in the treatment of gliomas were seen with Cilengitide, the study concluded that its action appeared to be antitumor cell specific as opposed to angiostatic [118]. Trials in other cancer patients failed to show any reduction in tumor load using Cilengitide [122, 123]. The low efficacy of the *α*V*β*3/*α*V*β*5 antagonists demonstrates that the mechanistic understanding of integrins in angiogeneses is not yet fully understood. At this time, antagonists for *α*V*β*3 or *α*V*β*5 alone do not appear to prevent angiogeneses, and other strategies need to be examined. Currently, integrin inhibitors for *α*2*β*1 [118, 124, 125] and *α*5*β*1 [118, 126] are being tested in phase I and phase II clinical trials.

#### 2.3.8. Cadherins

Cadherins are a class of calcium-binding transmembrane proteins that play an important role in cell-cell interactions. Several studies have underlined the important role of one particular cadherin, the vascular endothelial (VE) cadherin, in neovascularization. VE-cadherins are localized exclusively to the adherens junctions in ECs [127]. It has been suggested that VE-cadherins are important in regulating the passage of various molecules across the endothelium [28, 29]. In addition, VE-cadherin plays an important role in mediating EC growth through contact inhibition [48]. Mice deficient in VE-Cadherin showed extreme vascular abnormalities including diminished branching and sprouting, as well as disconnected ECs [48]. The vascular problems continued to progress until the vessels finally regressed or disintegrated [49]. This is thought to occur because the VE-cadherins establish EC junctional stability in the vessel walls. The cadherins also enhance EC survival by increasing the transmission of the antiapoptotic signal of VEGF [49]. Therefore, despite VE-cadherins non-existent role in vasculogenesis, it is vital to the maturation of blood vessels associated with angiogeneses [38].

Inhibition of VE-cadherins to prevent angiogeneses has been examined in several different animal models. Monoclonal Abs designed to recognize certain sections of extracellular repeats found in active VE-cadherins have been designed [128]. These Abs prevented EC junctional assembly and induced the disassembly of already existing EC junctions in vitro, abilities that could help prevent unwanted angiogeneses [128]. Although these original Abs were found to have some inhibitory effects on angiogeneses at low doses, significant vascular permeability was found in the heart and lungs of the mice at moderate to high doses, illustrating toxic side effects of the Abs [129, 130]. Since these original studies, other monoclonal Abs have been developed that do not exhibit the vascular permeability problems of the original version; these include BV14 and E4G10 [131, 132]. It is currently hypothesized that these second-generation monoclonal Abs will be effective because angiogenic junctions are weaker and contain different epitopes which are open to monoclonal Ab targeting [133]. Other groups have focused on the gene sequence of VE-cadherins, which may allow researchers to produce more specific monoclonal Abs for VE-cadherins and prevent unwanted angiogeneses [134]. 

VE-cadherins role in retinal neovascularization was also recently examined in mice. A group induced retinal neovascularization in newborn mice by exposure to oxygen [135]. Some of the mice were then treated with a VE-cadherin antagonist while others were treated with a control peptide. The mice treated with the VE-cadherin antagonist saw significantly reduced retinal angiogeneses compared to the control group [135]. In addition, the group treated with the antagonist had reduced levels of EC migration and proliferation as well as suppressed tubule formation from ECs [135]. As studies designed to better comprehend VE-cadherins role in angiogeneses have been completed, it has become clear that the role of cadherins in the angiogenic pathway is larger than just their adhesive activity. The ability of VE-cadherins to interact with various signaling molecules suggests that it has a role in EC growth, migration, survival, and morphogenesis [133]. Although VE-cadherin inhibitors alone are not capable of suppressing angiogeneses at this time, combining a VE-cadherin inhibitor with other inhibitors of angiogeneses may provide more complete suppression.

#### 2.3.9. Endoglin

Endoglin (Eng, CD-105) is a homodimeric cell surface glycoprotein that serves as a coreceptor for TGF-*β* [136]. Eng is found on proliferating ECs and also serves as an EPC marker [137]. It has been observed that Eng expression is greatly increased during angiogeneses and inflammation [138]. Studies have shown that Eng can regulate TGF-*β*, but the mechanism remains unknown [139]. With this function in mind, researchers have investigated the use of anti-Eng-based therapies in several different forms of cancer with the hopes of preventing tumor-based angiogeneses. Early in vitro studies using anti-Eng mAbs in the presence of human ECs showed that the mAbs greatly reduced growth of the ECs [140]. Following this work, in vivo studies showed that injections of anti-Eng mAbs into mice with colon or breast cancer xenografts demonstrated significant reductions in tumor size and had much greater survival rates than controls [141, 142].

The United States FDA approved a multicenter phase I clinical trial in 2008 using a naked anti-Eng mAB (TRC105) in patients with advanced and/or metastatic cancers [143]. Preliminary results from this trial have suggested clinical activity and tolerability of the mAb TRC105 in 17 patients [143]. As we await further results, no conclusive decision can be made about the clinical value of anti-Eng mAbs, though there does appear a reason for optimism. While this trial continues, several other groups have suggested conjugating the anti-Eng mABs with toxic molecules to ensure that the targeted ECs are killed. This model has had success in mice with breast cancer without any measurable toxicity [144]. In either case, Eng has been shown to be an important regulator of angiogeneses and a better understanding of its mechanistic course of action may help the drug design process in the future.

#### 2.3.10. Additional Factors

In addition to the factors mentioned above, many others have been shown to play important roles in angiogeneses, but their effects on the vasculature are not as widespread or as understood as the previously mentioned factors. An example of this is Tumor Necrosis Factor-*α* (TNF-*α*), a cytokine usually secreted by activated macrophages. TNF-*α* has been shown to help stimulate angiogeneses in vivo [50] and stimulate EC tube formation in vitro [51]. Transforming Growth Factor-*α* (TGF-*α*) is another cytokine secreted by macrophages and is capable of stimulating angiogeneses and EC proliferation in vivo [52]; however its role is still not completely understood. Angiogenin is another small polypeptide whose role in angiogeneses is still being investigated. It promotes angiogeneses in chorioallantoic membrane and rabbit cornea [53], but it is not mitogenic or chemotactic for ECs in vitro [53]. Angiogenin helps support EC adhesion and spreading in vitro [54]; however, its levels of synthesis are inconsistent with the timing of neovascularization in vivo [145]. Angiotropin is a polyribonucleopeptide originally isolated from peripheral monocytes [55]. Angiotropin is able to randomly induce capillary EC migration and tube formation in rabbit skin [55] and may trigger proliferative reactions in wound healing by activating microvascular ECs [38].

### 2.4. Angiogeneses Inhibitors: Current Research and Clinical Implications

#### 2.4.1. Angiostatin

Angiostatin is a 38 kDa internal fragment of plasminogen that displays inhibitory effects against tumor angiogeneses [146]. When plasminogen is in the vicinity of an implanted and/or primary tumor, it is cleaved by an unknown protease; a product of this cleavage is the antiangiogenic protein angiostatin [5, 147]. Research groups have shown that the endogenous protein is capable of inhibiting the growth of distant metastases [147, 148]. 

Experiments with angiostatin demonstrated that its activity leads to several different physiological results. As mentioned, angiostatin was shown to reduce the growth of remote metastasis [147]. This is accomplished by increasing the rate of apoptosis in metastatic tumors [149]. Apoptosis is increased because angiostatin attacks the energy system of the metastatic tissue by inhibiting ATP synthase F1F0, leading to caspase-mediated apoptosis [149, 150]. Another effect of activated angiostatin is the inhibition of capillary endothelial growth in vitro [151]. Mice with gliomas and melanomas experienced greatly reduced tumor growth and neovascularization when they were genetically engineered to express angiostatin [152, 153]. The activity of angiostatin was also examined on a global level using microarray techniques. A total of 189 genes had their expression levels altered with treatment of angiostatin. Most of these genes were involved in growth, apoptosis, and migration of ECs, as well as inflammation [6, 154], demonstrating the wide range of effects that angiostatin has within the body. 

To date, several clinical trials have tested the efficacy of angiostatin as a treatment for several forms of cancer. Subcutaneous injections of recombinant human angiostatin showed little to no toxicity in phase I clinical trials [155]. Phase II trials using angiostatin in combination with both paclitaxel and carboplatin have been completed in non-small-cell lung cancer (NSCLC) patients [156]. The response rate of the combinational therapy was higher than previous studies using the chemotherapy alone [156]. The group reported that the overall response rate to the combined treatment was 39.1%, another 39.1% of the patients remained stable, while the last 21.7% of the patients saw their disease progress [156]. Although the group did see improved rates of treatment, they fell below the expected levels. Several groups are working on alternate methods of administering angiostatin to increase its success as a treatment. Experiments using intravenous administration of angiostatin genes complexed to cationic liposomes are ongoing [157].

#### 2.4.2. Endostatin

Endostatin is an angiostatic 20-kD internal fragment of the carboxy terminus of collagen XVIII [151], an important proteoglycan in basement membranes. It was originally discovered in the blood and urine of tumor-bearing mice [151]. Two of the enzymes responsible for the release of endostatin include elastase [158] and cathepsin L [159]. Endostatin interacts with many different cell surface proteins including the integrins (*α*5*β*1 and to an extent also *α*V*β*3 and *α*V*β*5) [151] and several glycpians [160]. These interactions result in altered EC adhesion and migration [6]. 

In vitro, endostatin inhibits EC migration, proliferation, and tube formation [151], three key aspects of angiogeneses. Inhibition of angiogeneses via endostatin leads to a reduction of tumor growth in vivo [161]. It appears that this inhibition is partially accomplished by reducing the expression of VEGF [162]. Endostatin also has the ability to block existing VEGF from interacting with its receptor VEGFR-2 [163]. Endostatin reduces EC proliferation by arresting the EC cell cycle through the downregulation of cyclin-D1 promoter transcriptional activity [164]. As a result, the cell is unable to progress through the G1/S transition. Recent studies have also shown that endostatin disturbs the survival/death balance via activation of the proapoptotic pathway through the induction of caspase-9 activation [165]. These pathways induction is due to the endostatin-led decrease of the anti-apoptotic proteins Bcl-2, Bcl-Xl, and Bad [165]. Gene array and proteonomic analysis have given insight into the vast number of genes that can be affected by endostatin treatment in human dermal microvascular ECs. Approximately 12% of the 74,834 genes represented on the microarray chip had altered expression levels when treated with endostatin [166]. Both angiostatin and endostatin cause apoptotic pathway activation [166]. However, the identity and number of genes regulated by endostatin differ from angiostatin, suggesting alternative pathways of action. 

Evidence for endostatin's importance can been seen by studying individuals with Down Syndrome. People with Down Syndrome have a third copy of collagen XVIII due to a trisomy of chromosome 21. These individuals tend to have a 1.6–2 fold elevation of endostatin levels [167] and have greatly reduced levels of malignant tumors (except testicular cancer and megakaryocytic leukemia) [168], atherosclerosis [169], and diabetic retinopathy due to neovascularization [170]. These three diseases are all angiogeneses-dependent [5], and showcase the important role that endostatin may play in inhibiting unwanted pathogenic angiogeneses in humans. 

Endostatin is currently being analyzed for therapeutic potential in several forms of cancer. Using animal studies, a group recently demonstrated that it may be used as a possible treatment to boost the post-operative prognosis of osteosarcoma patients [171]. The study was designed to determine whether antiangiogenic treatment could help prevent the progression of pulmonary metastasis, a secondary problem often associated with postoperative osteosarcoma [171]. The group injected an adenovirus encoding endostatin vector (Ad5CMV-mEnd) two weeks after tumor inoculation [171]. The group found statistically significant differences in the size and prevalence of pulmonary metastasis between the control and treatment groups two weeks after the administration of the vector [171].

A recently completed animal study investigated the use of an endostatin-angiostatin fusion protein in Renal Cell Carcinomas (RCCs). The group tested the fusion protein's ability to inhibit tumor angiogeneses, tumor growth, and metastasis [172]. All animals underwent postmortem histopathological analysis of the liver, kidney, lung, spleen, and brain to determine levels of metastasis. The mice treated with the angiostatin-endostatin fusion protein had a 97% primary tumor growth reduction compared to the controls. In vivo tumor vascular imaging showed that the fusion-treated group had fewer blood vessels, and decreased lumen diameter [172]. These results indicate that sustained angiostatin-endostatin gene therapy may provide a novel treatment method for metastatic RCCs [172]. 

Clinical trials using endostatin for the treatment of several types of cancer are ongoing. Phase I clinical trials showed that endostatin is well tolerated by patients, but its antitumoral activity was minimal at best [173–175]. A multicenter phase II study of recombinant human endostatin use in carcinoid neuroendocrine tumors and pancreatic neuroendocrine tumors was recently completed. The endostatin vector was given subcutaneously to 42 patients with the advanced form of either disease [176]. None of these patients experienced significant toxicity; however they did not demonstrate a clinically relevant radiological response either [176]. Eighty percent of the patients receiving treatment experienced disease stabilization, while the other 20% had further disease progression [176]. Although the study found minimal benefit from the treatment, the group admitted that the optimal therapeutic dosage and form of administration are still unknown [176]. In addition, it has been suggested that adding endostatin to current chemotherapeutic strategies may enhance the efficacy of the treatment for carcinoid and pancreatic neuroendocrine tumors [176]. Another group examined the use of novel recombinant endothelial endostatin (YH-16) also known as “endostar,” for advanced NSCLC patients in a phase III trial [177]. The phase III trial treated one group of patients with endostar in combination with vinorelbine and cisplatin, while the other group of patients only received only vinorelbine and cisplatin [177]. The group that received endostar saw a response rate of 35.4%, while the group that received the chemotherapeutics alone saw response rates of only 19.5%. The median time to progression was 6.3 months in the endostar-treated group compared to 3.6 months in the control group. The clinical benefit rates for the chemotherapeutic and endostar-treated group were 73.3% while the rates for group receiving vinorelbine and cisplatin alone were 64% [177]. Overall, the study found that the addition of endostar to the vinorelbine and cisplatin treatment resulted in significant and clinically meaningful improvement compared to the vinorelbine and cisplatin treatment alone. 

#### 2.4.3. Tumstatin

Degradation of type IV collagen releases a 28 kDa fragment known as tumstatin, a compound that also displays antiangiogenic properties [178]. Tumstatin binds the *α*V*β*3 integrin, which results in G1 arrest and the induction of EC apoptosis [179]. Mice models have shown that exogenous tumstatin is able to inhibit the growth of tumors [179]. In addition, tumstatin-deficient mice had a much greater microvessel density near implanted murine tumors, and the mice had a 300% increase in overall tumor growth [5]. 

Animal studies determining the viability of tumstatin as an antiangiogenic-drug have been and continue to be completed. Tumstatin treated mice with teratocarcinomas showed over a 90% reduction in tumor size compared to controls [180]. The same group also examined a combination of anti-VEGF (Avastin) and tumstatin treatment compared to the tumstatin treatment alone. The animals receiving the combination treatment saw a statistically significant reduction in tumor growth when compared to the tumstatin alone or a placebo [180]. These findings demonstrated strong preclinical evidence for a future treatment of cancer with an anti-VEGF Ab alongside a tumstatin peptide [180]. Another recently completed animal study examined gene delivery of a tumstatin fragment into hepatocellular carcinomas (HCCs) [181]. In vivo intratumoral injection of the tumstatin fragment (pSecTag2B-tum-1) greatly diminished the growth of preestablished human HCCs [181]. In addition, there was a decrease in the amount of CD-34 positive vessels in the tumor [181].

#### 2.4.4. Platelet Factor-4

Platelet Factor-4 (PF-4) is a chemokine naturally secreted by platelets that normally promotes blood coagulation. In addition to this role, PF-4 is also known to be an inhibitor of angiogeneses. PF-4 is secreted from the alpha-granules of activated platelets and binds with high affinity to heparin-like glycosaminoglycans on the surface on ECs blocking them from further activity [6]. Studies have also shown that PF-4 blocks the upregulation of MMP-1 and MMP-3, inhibiting EC migration [182]. Finally, PF-4 is also capable of inhibiting the EC cell cycle by impairing pRB phosphorylation [183].

Despite the antiangiogenic and antitumoral effects of PF-4 in murine human tumor implant models [184], PF-4 has not proven to be an effective treatment for human cancers [185]. The early failure of PF-4 as a monotherapeutic treatment led researchers to examine different approaches for the use of PF-4 as an angiostatic agent. Recently, researchers produced a novel peptide containing the active fragment of PF-4 along with vasostatin, an inhibitor of EC proliferation [186]. This peptide was examined as a potential angiostatic agent in chick embryos. In vivo neovascular growth was compared between a group receiving the novel peptide, a group only receiving vasostatin, another receiving PF-4 alone, as well as a control. The chick embryos receiving the novel peptide showed a statistically significant reduction in angiogeneses when compared to the other groups [186]. Although the study was only completed in animals, it demonstrates another possible drug that may be used to inhibit tumor angiogeneses.

#### 2.4.5. Thrombospondin

Thrombospondin-1 (TSP-1), the first naturally occurring angiostatic protein discovered, is a multidomain matrix glycoprotein that has been shown to be a natural inhibitor of neovascularization [187]. Unlike previous angiostatic agents discussed, TSP-1 is a native, full-length protein. TSP-1 is stored in *α*-granules of platelets, where it is complexed with TGF*β*1 [188]. When released from the platelets and free from TGF*β*1, TSP-1 inhibits the migration of ECs [189] and induces EC apoptosis [190]. To slow migration, TSP-1 binds to EC surface receptors capable of promoting promigratory signals [189]. The induction of apoptosis in ECs is associated with TSP-1's ability to alter the concentrations of several important apoptotic factors. TSP-1 upregulates Bax, downregulates Bcl-2, and activates the caspase-3 intrinsic pathway, leading to programmed EC death [191]. Other experiments have shown that mice depleted of TSP-1 saw a 250%–300% increase in tumor growth rate when implanted with murine tumors [1]. Microvessel density was also increased near the tumor in these mice [5]. 

A phase I clinical trial using ABT-510, an angiogenic inhibitor derived from TSP-1, has been completed. This drug was delivered subcutaneously in patients with advanced solid malignancies. Although the phase I trial was not designed to test efficacy, 6 of the 39 patients saw disease stabilization after the treatment [192]. The toxicity effects associated with ABT-510 were minimal and deemed safe for future trials [192]. The use of ABT-510 alongside chemotherapeutic agents has also been examined. A phase I clinical trial investigated the use of ABT-510 with gemcitabine-cisplatin chemotherapy in patients with solid tumors [193]. This study found no clinically significant pharmacokinetic interactions between the combined drugs. Despite the low doses, 3 of the 12 patients tested saw a partial response with the drug treatment [193]. Another phase I trial examined the toxicity profile of ABT-510 along with the chemotherapeutics 5-fluorouracil and leucovorin [194]. Minimal toxicity was found by combining the drugs, and 4 of the 12 patients had tumor stabilization posttreatment [194]. Both of the combined phase I trials stated that the optimal dosage for ABT-510 has not been established yet, and determining these values would be important in future clinical trials.

#### 2.4.6. Tissue Inhibitors of Metalloproteinases

Tissue Inhibitors of Metalloproteinases (TIMPs) are a family of proteases, derived from cartilage, which inhibit MMPs. As previously mentioned, MMPs play an integral role in the initiation of angiogeneses. They are responsible for EC basement membrane degradation and EC remodeling [3, 67]. The newly formed ECM developed by MMPs during the angiogenic response provides a scaffold for ECs to adhere, migrate, and form tubes for nutrient delivery. The inhibition of MMPs by TIMPs reduces the angiogenic capacity of ECs [195, 196]. High levels of TIMP-1 greatly inhibit migration of ECs through gelatin in vitro [197]. The invasive potential, growth, and neovascularization of metastatic murine melanoma cells were inhibited in vivo when transfected with TIMP-2 [195]. 

The role of TIMP-3 in tumor angiogeneses was examined using mice models. The study found that TIMP-3 treatment of mice with lung cancer led to reduced angiogeneses in vivo through the inhibition of the VEGF-VEGFR-2 signaling pathway and the induction of EC-apoptosis [198]. The inhibition of this pathway and the EC associated death are in part due to the inhibition of MMP-2 by TIMP-3 [198]. Another study investigated the use of TIMP-1 gene transfer through an adenovirus as a way to treat established gastric cancer in nude mice. Compared to controls, mice transfected with TIMP-1 gene saw a significant reduction in the mean number of tumor vessels [199]. Although the exact mechanism of TIMPs inhibition of cancer progression remains unclear, TIMPs should still be considered for therapeutic trials because of their success in animal models.

#### 2.4.7. Interleukins

Interleukins (ILs) are a group of cytokines that are released by leukocytes and control a wide range of biological activities. A few of these ILs have been shown to affect the growth of blood vessels [200]. The ability to either enhance or suppress angiogeneses is based on a Glu-Leu-Arg (ELR) motif at the NH2 terminus. IL-8 possesses this sequence (discussed later), and therefore enhances angiogeneses, while IL-4 does not contain the motif, and is an inhibitor of angiogeneses [200]. 

IL-4 acts as an inhibitor of tumor growth [201], but its mechanism of action likely varies with different tumor cells. For example, IL-4 is thought to directly inhibit proliferation of cells from cancers such as colon tumors, head and neck tumors, and glioblastomas [202], while in other cases it is thought to induce a host immune response against the tumor cells such as in B-cell lymphomas and melanomas [203]. There is also evidence that IL-4 inhibits neovascularization, thus inhibiting tumor growth. In vitro, IL-4 inhibits migration of ECs towards bFGF. In vivo, IL-4 has been shown to inhibit neovascularization in rat corneas that should have been induced by the high concentration of bFGF present [204]. These experiments demonstrate that IL-4-mediated suppression of tumor growth may be due to IL-4s ability to inhibit angiogenic processes. Other noncancer-related animal studies have shown IL-4's antiangiogenic capabilities in vivo. One such study examined rats with adjuvant-induced arthritis. One set of animals received an adenovirus capable of producing IL-4, another group received a control virus without the IL-4 producing capabilities, and the last received a saline injection [205]. The group treated with the IL-4 producing adenovirus saw a statistically significant reduction in blood vessel growth [205]. The reduction in angiogeneses from the IL-4 treatment appears to be associated with a change in the pro- and antiangiogenic cytokine levels [205]. Although the study was designed to study inflammatory arthritis, knowledge gained about IL-4 could be used for the treatment of cancer in the future.

#### 2.4.8. Interferons (IFNs)

Interferons (IFNs) belong to a large family of secreted glycoproteins known as cytokines. They are produced and secreted by a wide variety of immune-related cells and IFN-*α* has been shown to inhibit angiogeneses in vivo [206]. It is thought that both IFN-*α* and IFN-*β* are able to inhibit angiogeneses by repressing bFGF mRNA and protein levels [207]. In addition to the downregulation of key angiogeneses signaling factors, IFN-*α* also inhibits the migration of EC cells in vivo [208].

Several clinical trials have been performed investigating the administration of IFN-*α* in combination with chemotherapeutics in different forms of cancer. Many studies have used PEG IFN-*α*, a form of IFN-*α* that has been modified to have a longer half-life and thus have a greater opportunity to reduce angiogeneses [209]. A phase I trial completed tested the effect of subcutaneous injections of PEG IFN-*α* and recombinant IL-2 in patients with metastatic RCC [210]. Minimum toxicity was found at most levels, and an unsafe level of the drug combination was determined. Of the 34 patients in the study, 15% saw a partial response, 68% had disease stabilization, and 18% had their disease continue to progress. Overall, the median survival was 31.9 months for patients treated and their median progression-free survival was 9.0 months [210]. Both the median survival time and the median progression-free survival time were slightly greater than the average control reported in the literature [210]. It was determined from this study, though, that IFN-*α* alone or in combination with IL-2 is not the optimal treatment of RCCs. The group did suspect that the use of IFN-*α* in combination with Avastin (a monoclonal Ab that targets VEGF) may provide a better treatment for RCC [210]. This theory was tested in a clinical trial using patients who also had metastatic RCCs. They reported that the median progression-free survival in patients receiving the combined Avastin and IFN-*α* treatment was double that of patients only receiving IFN-*α* and a placebo [211]. The combined therapy also showed improvement in overall response rate from 13% in the IFN-*α* alone group to 32% in the combined treatment arm of the study [211]. A clinical benefit was seen in 79% of the patients receiving combined treatment versus 65% in the IFN-*α* alone treatment group [211]. Lastly, the median duration of tumor response was 13.5 months in the combined drug group while it was only 11.1 months in the IFN-*α* only group [211]. After completion of the study, it was observed that the levels of IFN-*α* used were higher than the optimal dosage, and it is likely that treatment with slightly lower levels of IFN-*α* would have seen greater clinical benefit in both groups [211]. The study determined that the combination therapy was an effective first-line treatment option for metastatic RCC [211], and further trials should be conducted to determine optimal dosages.

In addition to metastatic RCCs, clinical trials have also tested the use of PEG IFN-*α* as a treatment for metastatic melanomas. A phase I trial using PEG IFN-*α* determined that it was safe and effective in humans [212]. A phase II trial then investigated the use of Dacarbazine (DTIC) alongside PEG IFN-*α* for the treatment of metastatic melanomas. Of the 25 patients who completed the study, 8% had complete remission from the treatment, while another 16% had partial responses [209]. Stable disease was found in 4% of patients while 72% had their disease progress. Although the treatment proved that the combinational treatment was not toxic, the efficacy of the treatment could not be determined because the study did not compare different regimens of treatment [209]. Despite this, the 24% overall response rate, including two long-term complete responses, is promising and warrants more clinical investigation [209].

#### 2.4.9. Tumor Angiogeneses

Tumors begin as an avascular mass of host-derived cells that proliferate atypically because they have lost the ability to control their growth [3]. Tumors initially survive and thrive on vasculature that is already available in the surrounding host environment [36]. In order for tumors to grow beyond 2-3 mm^3^, they need a continual supply of blood to remove waste and deliver nutrients [1]. Hypoxia of tumor cells will occur if the tumor grows beyond the maximum distance of effusion from local vessels (around 200 *μ*m) [36]. In order to counter this lack of oxygen, tumor cells will attempt to create new blood vessels to supply their needs in a mechanism that closely resembles normal angiogeneses [3]. This process and several important tumor-derived factors are illustrated in Figure 3. 

The blood vessels formed during tumor-induced angiogeneses are abnormal. The walls of tumor vessels are usually made of a combination of both tumor cells and ECs [213]. Functional pericytes are often absent from the peripheral blood vessels [214], leaving an incomplete basement membrane. This causes those vessels to be especially leaky and dilated [215]. Recently, it was found that, although the majority of tumor vasculature does not possess pericytes, some tumors keep a core of blood vessels alive and functional because the vessels are protected by pericytes [216, 217]. This concept emerged from several studies that showed VEGF signaling inhibition led to a large reduction in tumor vascularity. However, functional vessels that remained after treatment were small in diameter and covered with pericytes [216, 218]. The morphology of these surviving tumor vessels was very different from normal dilated tumor vessels which are sparsely populated with pericytes [216, 218]. These observations support the prevailing thought that tumor vessels lacking pericytes are more vulnerable to anti-VEGF treatment [218, 219].

Like the normal angiogenic process, tumor angiogeneses is reliant on VEGF and other angiogenic proteins. Increased levels of VEGF and its receptor VEGFR-2 have been observed in many cancers, including metastatic human colon carcinomas, where increased levels were shown to directly increase tumor vascularization [220]. Breast cancer patients with higher levels of VEGF expression have increased intratumoral vascularization and a worse prognosis [221]. Experiments with monoclonal Abs against VEGF, or genetic inactivation of VEGF (or VEGFR-2), have dramatic decreases in angiogeneses and neovascularization in several different forms of cancer [83–85]. VEGF is stimulated by the hypoxic conditions near the central necrotic tissue of solid tumors [222]. The mechanism of tumor blood vessel growth activated by VEGF is similar to the normal angiogenic response to hypoxic conditions. Neovascularization occurs in both cases to help meet the metabolic needs of cells [2, 3]. In addition to the endovascular stimulation attributed to VEGF, it can also increase vascular permeability [28, 29, 223, 224], explaining the leaky blood vessels observed in tumors. VEGF has many important roles in tumor angiogeneses and therefore its inactivation has often been a target of tumor therapy.

Ang-2 plays a more important role in tumor angiogeneses than it does in normal angiogeneses. As an antagonist for Ang-1, it is largely responsible for blood vessel destabilization seen in vasculature surrounding tumors. Normally, destabilization leads to blood vessel breakdown, but in the presence of VEGF, the vasculature is readily receptive to VEGF-mediated growth [225]. Ang-2 expression in ECs of tumor vessels greatly exceeds that of ECs in normal blood vessels and can be used as an early biomarker of tumor-induced vascularization [226]. Besides giving the growing vasculature plasticity in the presence of VEGF, it also plays an important role in the initial stages of tumor angiogeneses. During early tumor development, VEGF levels are greatly reduced, but Ang-2 mRNA levels are high [227]. At this stage, Ang-2 is responsible for the degradation and regression of blood vessels associated with early stages of tumor angiogeneses. As the tumor continues to grow in size, it eventually reaches a point where it requires more nutrients, thus levels of VEGF rise and new capillary growth can begin.

FGF was the first tumor-derived factor found to stimulate neovascularization and EC proliferation in vivo [228]. The importance of bFGF in tumor angiogeneses was confirmed by the use of bFGF receptor inhibition in tumor-injected mice. Inactivation of bFGF receptor led to decreased tumor growth [228] and blood vessel density [229]. Based on the time that bFGF is active during tumor angiogeneses, it has been suggested that bFGF is important in maintaining this process, as opposed to VEGF which likely initiates tumor angiogeneses [229]. However, bFGF has also been shown to help increase VEGF production [230], upregulate VEGF mRNA in vascular smooth muscle [231], and increase VEGF receptor density in microvascular ECs [232]. 

It was recently discovered that TGF-*β* signaling behaves as a strong activator of tumor growth and metastasis through stimulation of angiogenic processes [233, 234]. It is believed that TGF-*β* expression by neoplastic cells acts to induce the stromal reaction, which results in the formation of a reactive stroma microenvironment that is thought to promote angiogeneses and tumor growth [235]. It has also been shown that the use of neutralizing Abs against TGF-*β* leads to a reduction in the amount of blood vessels surrounding implanted tumors and greatly inhibits angiogeneses in these regions [235].

Heparanase is highly reactive 50 kDa protein known to induce tumor angiogeneses [236]. Heparanase is preferentially expressed in both melanoma and carcinoma [237]. Transfection of both nonmetastatic T lymphoma and melanoma cell lines with the heparanase gene caused both cell lines to become highly metastatic in vivo [236]. In addition, T lymphoma cells transfected with the heparanase gene saw a considerable increase in neovascularization near implanted tumors when compared to the nontransfected T lymphoma cells [236]. Heparanase stimulates angiogeneses directly by promoting EC invasion and vascular sprouting [3]. Heparanase also helps release bFGF that is bound to heparin sulfate at the ECM [236], increasing local bFGF levels. The increase in the local bFGF concentration is thought to contribute to the increased neovascularization measured near implanted tumors. 

Interleukin-8, which is produced by macrophages, is not an important factor in normal angiogeneses. However, IL-8 appears to be a central mediator of tumor-derived angiogeneses. Elevated levels of IL-8 have been documented in several types of neoplastic tissues [238]. The increased expression of IL-8 correlates with amplified neovascularization density [238, 239] as well as an increase in tumor growth [238]. Melanoma cells forced to continually express IL-8 were highly tumorigenic and had greater metastatic potential compared with parental and control transfected cells [238]. An important characteristic of IL-8 is its ability to increase levels of MMP-2 which degrades the EC basement membrane and remodels the ECM, initiating the early phase of tumor angiogeneses [21]. IL-8-transfected melanoma cells displayed greatly increased levels of MMP-2, while transfection of identical melanoma cells with VEGF and bFGF did not affect MMP-2 levels [240, 241] demonstrating that this is an important but separate mechanism involved in tumor-induced angiogeneses.

It is obvious that many different factors play an important role in tumor angiogeneses. To date, VEGF has been shown to play the most dominant role, but many other factors such as IL-8, MMP-2, heparanase, TGF-*β*, and bFGF also play an essential part in the process. Because so many factors are involved with tumor angiogeneses, it is likely that several of these factors must be inhibited simultaneously in order to significantly reduce the unwanted angiogeneses and eventual tumor metastasis.

## 3. Future Directions

Currently, investigation into mechanisms of angiogeneses inhibition in cancer is an important and promising area of research. Prohibiting angiogeneses is an important therapeutic approach for fighting cancer, reducing atherosclerosis, and preventing blindness due to retinal neovascularization in diabetic patients. In recent years, several new angiostatic therapies have been tested and approved by the FDA; examples include Avastin, Tarceva, and Lucentis [242]; several others are currently being tested in phase III trials throughout the country. Among them are possible angiostatic treatments for many different types of cancer including esophageal cancer, pancreatic cancer, lymphoma, renal cell cancer, gastric cancer, and many others [82]. Important advances have also taken place in defining the molecular understanding of angiogeneses. This includes a greater understanding of both angiogeneses as a whole as well as the mechanism of antiangiogenic drugs currently being used. However, the many studies using anti-VEGF treatments illustrate that our knowledge of the angiogenic pathway remains incomplete. In the past, it was thought that angiostatic treatment would create a form of cancer treatment that would evade the problem of resistance [243, 244]. As clinical trials continue, it now appears that many tumors can overcome the use of angiogenic inhibitors, thereby acquiring a way to bypass the therapeutic angiogeneses blockade [9, 82, 90]. Although there are several different adaptive mechanisms that tumors may employ to overcome antiangiogenic therapy, two concepts have emerged as the most likely candidates [82]. The first is that tumors are able to activate or upregulate alternative proangiogenic pathways after the first pathway is inhibited. An example was observed in animal models when a monoclonal Ab that specifically blocked VEGFR signaling was used in mice with tumors. The mice saw an initial response to the treatment, and the tumors possessed reduced vascularity [245]. After a short period of time, however, the tumors saw a reinitiation of tumor angiogeneses. When the tumors were resected and studied, they were found to express greater levels of mRNA for the proangiogenic factors FGF and Ang-1. This change in expression helps explain one possible method the tumors are employing to overcome VEGF inhibition. To further test the effect of upregulation of alternative proangiogenic factors, a similar study was conducted in which some mice were treated with VEGF inhibitor alone while others were treated with a VEGF inhibitor as well as an FGF trap. Mice treated with combinational therapy saw a great reduction in vascularization and slowed tumor growth [245]. Yet another study in mice showed that the induction of IL-8 was able to maintain angiogenic capability in tumors that did not express HIF-1*α*, an inducer of VEGF expression [246]. This alternative pathway illustrates another proangiogenic pathway that tumors may use to increase vascularization. Together, these studies have begun to shed light on why anti-VEGF treatments alone may have seen limited results in clinical trials. 

The other theory to why tumors have been able to withstand the anti-VEGF treatment is that resistant tumors have increased pericyte support on their tumor vasculature. The pericytes are believed to protect the remaining vessels and defend against the anti-VEGF treatment [219, 247]. The hypothesis states that tumor pericytes are most likely expressing appreciable levels of VEGF and possibly other proangiogenic factors [247]. In addition, pericytes are capable of reducing the rate of EC proliferation which allows EC maturation and stabilization in newly formed blood vessels [248]. Currently, several ongoing clinical trials are attempting to prevent tumor angiogeneses by inhibiting pericyte association with tumor vasculature along with angiogenic factors [82, 219]. 

These two ongoing theories showcase the current problems in the field of antiangiogenic research in cancer. After several years of clinical trials, it appears that targeting one angiogenic factor is not enough to permanently halt neovascularization in most tumors. Although these results were initially disheartening, they also opened up the possibility of other angiostatic therapies. Many clinical trials now use existing chemotherapeutic drugs or radiation along with antiangiogenic drugs. This two-front attack has had more success than antiangiogenic drugs or chemotherapy alone in a majority of patients [5, 118, 249]. 

As research continues, more information is also being uncovered about the angiogenic pathway. Increased understanding of the angiogenic pathway will allow for development and use of drugs that can target several angiogenic factors concurrently, allowing greater inhibition of angiogeneses, and increasing the likelihood of therapeutic success. Although the benefit of antiangiogenic treatments has not been as great as initially anticipated, many advances have come from their development and clinical use. However, with time, it is likely that the success of angiogenic treatment in cancer will continue to improve and we will come ever closer to the original goal of curing cancer and other angiogenic-related diseases.

## Figures and Tables

**Table 1:** Factors regulating angiogenesis.

Angiogenesis factors	Biological activities
Vascular endothelial growth factor (VEGF)	(i) Promoter of angiogeneses and vasculogenesis [21] (ii) Stimulates microvascular EC proliferation [22, 23] (iii) Enhances EC migration and sprouting [24, 25] (iv) Inhibits EC apoptosis [26] (v) Increases EC permeability [27–29]

Fibroblast growth factor (FGF)	(i) Stimulates EC proliferation [30] (ii) Promotes microvessel tube formation [30] (iii) Promotes EC migration [30] (iv) Important promoter of blood vessel remodeling after tissue injury [31]

Angiopoieten-1 (Ang-1)	(i) Recruits pericytes to recently created blood vessels [32] (ii) Helps promote EC survival and sprout formation [32, 33] (iii) Increases the diameter of blood vessels endothelium [34]

Angiopoieten-2 (Ang-2)	(i) Antagonist of Tie-2 receptor, reduces levels of pericytes [35] (ii) Increases plasticity of newly formed blood vessels [36]

Platelet-derived growth factor (PDGF)	(i) Increases capillary wall stability [3] (ii) Stimulates the proliferation of cultured pericytes and SMCs [37] (iii) Increases DNA synthesis on capillary ECs [38] (iv) Stimulate formation of angiogenic sprouts in vitro [38]

Transforming growth factor-*β* (TGF-*β*)	(i) At low doses upregulates angiogenic factors and proteinases [39] (ii) At high doses, inhibits EC growth, promotes reformation of BM and stimulates SMC reformation [39] (iii) Stimulates or inhibits EC tube growth [39] (iv) Signals inflammatory mediators such as fibroblasts and monocytes [3, 40, 41] (v) Enhances integrity of vessel walls [42]

Integrin *α*V*β*3	(i) Binds and activates MMP2 to help break down ECM [43] (ii) Helps regulate cell attachment, spreading, and migration [44] (iii) Shows Increased activity near wound sites [45] (iv) Localized to ECs at ends of growing vessels during EC sprouting [46]

Integrin *α*V*β*5	(i) Interacts with VEGF to promote angiogeneses [47]

VE cadherin	(i) Thought to mediate passage of molecules across endothelium [28, 29] (ii) Regulates CD growth through contact inhibition [48] (iii) Helps prevent EC apoptosis by promoting VEGFs signal [49] (iv) Helps stabilize the branches and sprouts produced during angiogeneses [48]

Tumor necrosis factor-*α* (TNF-*α*)	(i) Stimulates angiogeneses in vivo [50] (ii) Stimulates EC tube formation in vitro [51]

Transforming growth factor-*α* (TGF-*α*)	(i) Promotes EC proliferation [50] (ii) Stimulates angiogeneses in vivo [50, 52]

Angiogenin	(i) Promotes angiogeneses in vivo [53] (ii) Assists EC adhesion and spreading in vitro [54]

Angiotropin	(i) Helps activate microvascular ECs during wound healing [38] (ii) Stimulates angiogeneses in vivo [38] (iii) Randomly induces capillary EC migration [55]

Matrix metalloproteinase-9 (MMP-9)	(i) Thought to help mobilize EPCs by cleaving ECM [17]

Stromal-cell-derived factor-1 (SDF-1)	(i) Helps guide EPCs to ischemic areas during angiogeneses [56]

## Abbreviations

Ang: Angiopoieten
Ab: Antibody
AMD: Age related macular degeneration
CD: Cluster of differentiation
Eng: Endoglin
EC: Endothelial cell
EPC: Endothelial progenitor cell
ECM: Extra cellular matrix
FDA: Food and drug administration
FGF: Fibroblast growth factor
HSC: Hematopoietic stem cell
HCC: Hepatocellular carcinoma
HUVEC: Human umbilical vein endothelial cell
HIF: Hypoxia inducible factor
IFN: Interferon, IL-interleukin
MMP: Matrix metalloproteinase
mAB: monoclonal Antibody
MAPC: Multipotent adult progenitor cell
NSCLC: Non-small-cell lung cancer
PDGF: Platelet derived growth factor
PF: Platelet factor, rcc-renal cell carcinoma
RCC: Renal cell carcinoma
TSP: Thrombospondin
TIMP: Tissue inhibitor of metalloproteinase
TGF: Transforming growth factor
TNF: Tumor necrosis factor
VE: Vascular endothelial
VEGF: Vascular endothelial growth factor
VWF: von Willenbrand factor.
